# Supplementary figures and images for: The Pattern Recognition Receptor FLS2 Can Shape the Arabidopsis Rhizosphere Microbiome β-Diversity but Not EFR1 and CERK1
Source: Plants (Basel). 2022 May 17;11(10):1323. doi: 10.3390/plants11101323 (PMC9147754; doi:10.3390/plants11101323)

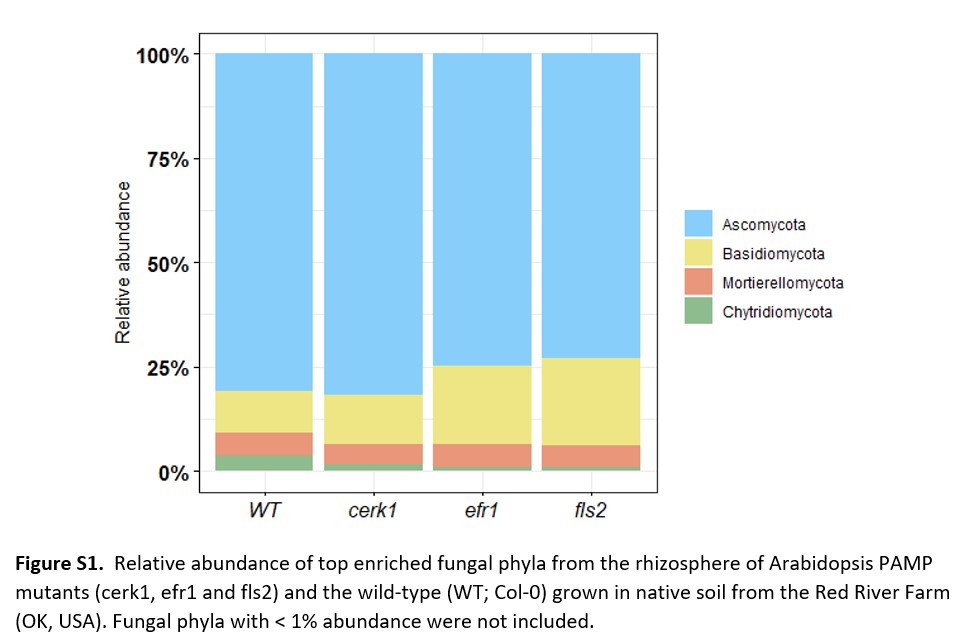

Supplement: Supplementary file 1 [file plants-11-01323-s001.zip › Figure S1.jpg]
